# The effect of low-intensity whole-body vibration with or without high-intensity resistance and impact training on risk factors for proximal femur fragility fracture in postmenopausal women with low bone mass: study protocol for the VIBMOR randomized controlled trial

**DOI:** 10.1186/s13063-021-05911-4

**Published:** 2022-01-06

**Authors:** Belinda Beck, Clinton Rubin, Amy Harding, Sanjoy Paul, Mark Forwood

**Affiliations:** 1 Menzies Health Institute Queensland, School of Health Sciences and Social Work, Griffith University, Gold Coast, QLD Australia; 2 Department of Biomedical Engineering, State University of New York at Stony Brook, New York, NY USA; 3 Melbourne EpiCentre, University of Melbourne and Melbourne Health, Melbourne, VIC Australia; 4 School of Pharmacy and Medical Sciences, Gold Coast, QLD Australia

**Keywords:** Exercise, Fracture, HiRIT, Osteoporosis, Postmenopausal women, Vibration

## Abstract

**Background:**

The prevailing medical opinion is that medication is the primary (some might argue, only) effective intervention for osteoporosis. It is nevertheless recognized that osteoporosis medications are not universally effective, tolerated, or acceptable to patients. Mechanical loading, such as vibration and exercise, can also be osteogenic but the degree, relative efficacy, and combined effect is unknown. The purpose of the VIBMOR trial is to determine the efficacy of low-intensity whole-body vibration (LIV), bone-targeted, high-intensity resistance and impact training (HiRIT), or the combination of LIV and HiRIT on risk factors for hip fracture in postmenopausal women with osteopenia and osteoporosis.

**Methods:**

Postmenopausal women with low areal bone mineral density (aBMD) at the proximal femur and/or lumbar spine, with or without a history of fragility fracture, and either on or off osteoporosis medications will be recruited. Eligible participants will be randomly allocated to one of four trial arms for 9 months: LIV, HiRIT, LIV + HiRIT, or control (low-intensity, home-based exercise). Allocation will be block-randomized, stratified by use of osteoporosis medications. Testing will be performed at three time points: baseline (T0), post-intervention (T1; 9 months), and 1 year thereafter (T2; 21 months) to examine detraining effects. The primary outcome measure will be total hip aBMD determined by dual-energy X-ray absorptiometry (DXA). Secondary outcomes will include aBMD at other regions, anthropometrics, and other indices of bone strength, body composition, physical function, kyphosis, muscle strength and power, balance, falls, and intervention compliance. Exploratory outcomes include bone turnover markers, pelvic floor health, quality of life, physical activity enjoyment, adverse events, and fracture. An economic evaluation will also be conducted.

**Discussion:**

No previous studies have compared the effect of LIV alone or in combination with bone-targeted HiRIT (with or without osteoporosis medications) on risk factors for hip fracture in postmenopausal women with low bone mass. Should either, both, or combined mechanical interventions be safe and efficacious, alternative therapeutic avenues will be available to individuals at elevated risk of fragility fracture who are unresponsive to or unwilling or unable to take osteoporosis medications.

**Trial registration:**

Australian New Zealand Clinical Trials Registry (www. anzctr.org.au) (Trial number ANZCTR12615000848505, https://www.anzctr.org.au/Trial/Registration/TrialReview.aspx?id = 368962); date of registration 14/08/2015 (prospectively registered). Universal Trial Number: U1111-1172-3652.

**Supplementary Information:**

The online version contains supplementary material available at 10.1186/s13063-021-05911-4.

## Background

Osteoporosis is defined as a progressive, systemic skeletal disease characterized by profound loss of bone mass and microarchitectural deterioration, the consequence of which is increased bone fragility and risk of fracture [1]. Although either sex can be affected, osteoporosis is considerably more common in women than men. Virtually any bone can be affected, but fractures occur most often at the vertebrae and distal forearm [2]. Proximal femur (“hip”) fractures cause the greatest personal and economic burden. Adverse sequelae of hip fractures include severe pain, disability, hospitalization, surgery, and loss of independence, all of which have substantial negative impact on quality of life [2]. The economic impact of hip fractures is substantial and rising. In Australian women aged 50 and over, the total direct costs equated to $734 million in 2017 [3]. According to Watts and colleagues (2013), the prevalence of osteopenia and osteoporosis in older women (i.e., 50 years of age) will increase from 2.62 million in 2012 to 3.44 million in 2022, with a commensurate 25% increase in hip fractures in this population [4]. These figures highlight the burden of poor bone health in older women, and the need for effective prevention strategies.

The primary therapeutic approach to manage osteoporosis and fracture prevention is pharmacotherapy, comprising antiresorptive (bisphosphonates, denosumab, hormone therapy) and anabolic (teriparatide and romosozumab) agents, which have varying degrees of antifracture efficacy [5]. A proportion of individuals fail to respond to osteoporosis medications, suffering incident fracture or continued loss of bone mass over time [6]. Furthermore, over 90% of hip fractures are a direct result of a fall [7] and medications do not reduce falls or even improve important risk factors for falling such as muscle strength and balance. It is perhaps unsurprising then that osteoporosis medication uptake is low and adherence (compliance and persistence) decreases over time [8, 9]. Despite an increasing prevalence of osteoporosis, adoption of medication dropped by 15% in Australia from 2007 to 2014 [10], a likely consequence of a number of severe, albeit rare, adverse effects [11] that received considerable media attention. Given the scale and burden of osteoporotic fracture, the limitations of pharmacotherapy and the reluctance of many patients to take them, the identification of effective non-pharmacological strategies for the prevention and management of osteoporosis is pressing.

Traditionally, exercise has been considered only an ancillary strategy to prevent osteoporotic fracture. The inherent fragility of an osteoporotic skeleton typically motivates clinicians to recommend exercise at only low intensity to avoid load-related fracture. Unfortunately, low-intensity exercise is rarely sufficient to increase bone mass and strength [12, 13]. While animal studies confirm that high-magnitude loads are necessary to stimulate bone accretion [14], the translation to clinical practice has been hampered by safety concerns. Recently, however, a series of trials have challenged this reticence. The LIFTMOR trial for postmenopausal women with low bone mass, and the LIFTMOR-M trial for older men (also with low bone mass), found that high-intensity progressive resistance and impact training (HiRIT) improved bone mass and other risk factors for falls and fracture, with no serious adverse events [15, 16]. Furthermore, the HiRIT program improved thoracic kyphosis and was not associated with an increased risk of incident vertebral fractures [17, 18].

It has also been observed from animal studies that light loads applied at sufficiently fast rates, in the order of 30 Hz, can also enhance bone formation [19]. As it is not physically possible to move the body fast enough to apply loads to the skeleton at 30 Hz, low-intensity whole-body vibration (LIV) devices were developed to do so passively. If effective, LIV may be an appealing osteoporosis therapy for individuals unwilling or unable to exercise at high loads. Extensive work in mice [20], rats [21], turkeys [22], sheep [19], and humans [23] has shown LIV can normalize bone endpoints in human and animal models of disease [24]. In mice, LIV improved disuse-associated bone loss [25], restored bone lost prior to reambulation [21, 26], and regenerated periosteal bone subsequent to surgery [27] by suppressing bone resorption and increasing bone formation [28]. In humans, LIV (30 Hz at 0.3 to 0.4 *g*) enhanced bone density in children with cerebral palsy [29, 30], Duchenne muscular dystrophy [31], and scoliosis [30]. LIV has been observed to reduce bone loss in patients with osteoporosis [32], chronic obstructive pulmonary disease [33], and young women with low bone mineral density (BMD) [34].

Results from vibration trials in postmenopausal women have been equivocal with discrepancies potentially due to vibration stimulus heterogeneity (e.g., frequency, magnitude, and cumulative dose) and inconsistent use of control groups (e.g., inactive control group or sham/placebo vibration). It has been suggested that vibration enhances bone mass only at certain sites [35, 36], or reduces falls but has no effect on bone mass in older adults [37]. The single pilot trial comparing whole-body vibration (WBV) with exercise training in postmenopausal women showed vibration (amplitude 1.7–2.5 mm, frequency 35–40 Hz) improved total hip (TH) BMD more than moderate-intensity resistance training [38]. The single trial examining the combined effect of WBV and exercise on falls and fracture risk parameters reported no difference in effect of WBV plus exercise versus exercise alone [39]. The safety of the high-intensity vibration stimulus employed in the latter trials for the osteoporotic demographic is of some concern. No trial has conducted a head-to head comparison of LIV with or without a known efficacious exercise protocol (i.e., HiRIT) on indices of hip fracture risk in postmenopausal women with low bone mass.

Therefore, the overarching aim of the “Vibration and exercise Intervention for Bone, Muscle and Osteoporosis Rehabilitation” (VIBMOR) trial is to determine the effects of 9 months of 5 days per week, 10 min, LIV (0.4 *g*, 30 Hz) on risk factors for hip fracture in postmenopausal women with low to very low bone mass, with or without twice-weekly, 30 min, bone-targeted HiRIT. Intervention responses will be compared with a 9-month, twice-weekly, 30 min, home-based, low-intensity exercise program known to be an ineffective bone stimulus (a.k.a. active control). We will also explore whether any effects are maintained after detraining (removal of stimulus). Quality of life and cost-effectiveness will also be examined. We hypothesize that the combination of LIV and HiRIT will improve primary and secondary outcomes more than either therapy alone (that is, LIV or HiRIT), or control.

## Methods

### Human research ethics approval and trial registration

The VIBMOR study has been granted approval from the Griffith University Human Research Ethics Committee (Protocol number: AHS/61/14/HREC; Human Research Ethics Committee Reference: 2014/828). All research activities will be conducted in accordance with the *Declaration of Helsinki*, the NHMRC National Statement on Ethical Conduct in Human Research (2007), the Notes for Guidance on Good Clinical Practice as adopted by the Australian Therapeutic Goods Administration (2000) (CPMP/ICH/135/95), and the ICH Good Clinical Practice Guidelines. The study is registered with the Australian New Zealand Clinical Trials Registry (Trial number ANZCTR12615000848505; prospective registration). Written informed consent will be obtained by research staff from all participants deemed eligible for the trial during preliminary screening prior to the investigator performing any baseline assessments. A copy of the VIBMOR trial consent form can be provided upon request.

### Study aims

The primary aim of the VIBMOR trial is to compare the efficacy of LIV, alone and in combination with HiRIT, with and without osteoporosis drugs, to improve risk factors for hip fracture in postmenopausal women with low to very low bone mass. While fracture per se is the outcome of greatest clinical concern, it has been projected that adopting a hip fracture endpoint in an exercise trial would require a sample size so large (approximately *n* = 14,000) as to be unfundable and is therefore unlikely to ever be attempted [40]. DXA-derived aBMD is strongly negatively associated with fragility fracture in women, thus the application of aBMD as a surrogate endpoint for fracture in osteoporosis research is justified [41] and routinely adopted. The practice has recently been statistically validated through meta-regression [42, 43]. Therefore, in the proposed trial, intervention efficacy will be assessed based on known risk factors for hip fracture: primarily, TH aBMD as well as other indices of bone strength. Our secondary outcomes will include falls and fall risk factors through indices of muscle strength, dynamic and static balance, and physical function [44]. Exploratory outcomes will provide preliminary insight into mechanisms (bone turnover markers), and potential ancillary benefits (pelvic floor health and dysfunction, fracture, quality of life), feasibility (compliance, physical activity enjoyment), and safety (adverse events) of the interventions. Analyses will be adjusted for presence or absence of osteoporosis medication, intervention compliance, dietary calcium, and serum vitamin D.

### Study design and description

**Fig. 1:**
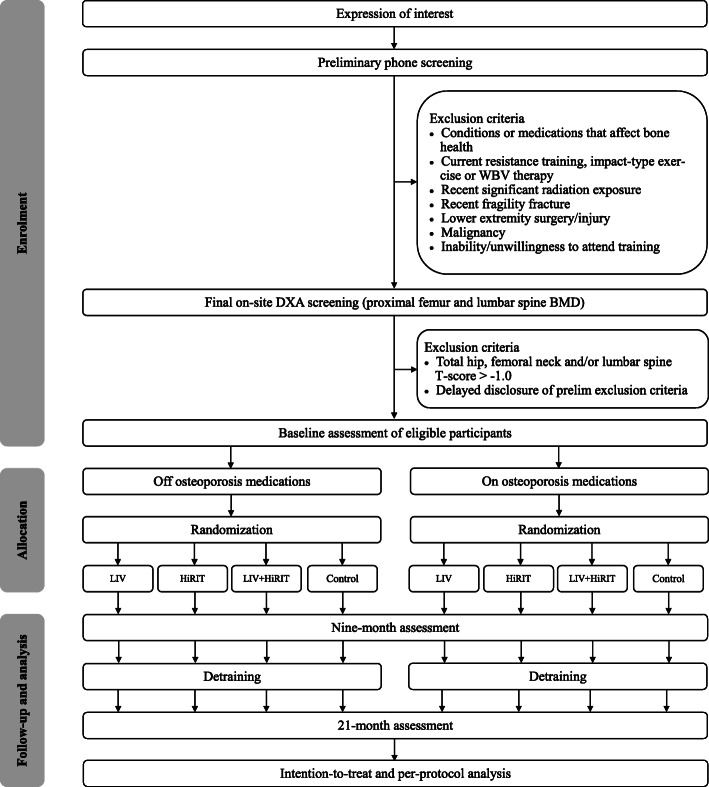
The CONSORT diagram: proposed participant flow

VIBMOR is a single-center, 21-month, four-arm, single-blind, block-randomized controlled trial. The trial is divided into two phases, a 9-month treatment intervention followed by 1-year withdrawal of treatment to monitor detraining effects. The Consolidated Standards of Reporting Trials (CONSORT) diagram of proposed participant flow through the trial is illustrated in Fig. 1, and the Standard Protocol Items: Recommendations for Interventional Trials (SPIRIT) schedule of enrollment, interventions and assessments is illustrated in Table 1. The 9-month exercise intervention period has been chosen as the minimum time frame in which notable changes to bone mass are likely to be detected from densitometry [45].

**Table 1:**
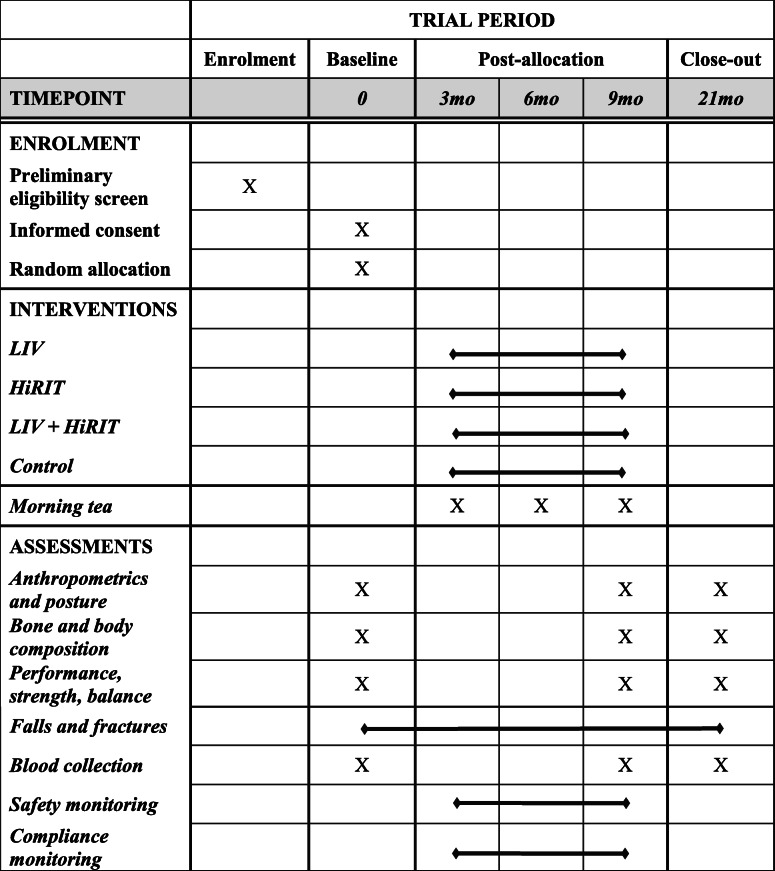
The SPIRIT Schedule: VIBMOR trial schedule of enrolment, interventions, and assessments

Baseline and follow-up assessments will be conducted in the Bone Densitometry Research Laboratory, Griffith University, Gold Coast campus, Queensland, Australia. All supervised training sessions will be conducted in the Strength Training Research Facility, co-located in the School of Health Sciences and Social Work. All trial-related activities, measurements and interventions will be conducted at this single location (Griffith University, Gold Coast, Queensland, Australia).

Participants will be postmenopausal women with low aBMD (TH, femoral neck [FN] and/or LS aBMD *T*-score ≤ − 1.0 [46]) with or without previous fragility fracture. Participants will be randomly allocated to one of four groups: LIV, HiRIT, LIV + HiRIT, or control. Group allocation will be block-randomized according to use of osteoporosis medications as follows: (1) have not been taking any osteoporosis medications for the preceding 12 months and do not intend to start taking any such drug in the subsequent 21 months, and (2) have been taking antiresorptive osteoporosis medications for the preceding 12 months and intend to continue taking the same drug in the subsequent 21 months.

Testing of all outcome measures will be performed at three time points, baseline (T0), 9 months (T1), and 21 months (T2). LIV, HiRIT, and control group compliance will be monitored across the 9-month intervention period, while safety (adverse events and injuries), healthcare utilization, falls, and fractures will be monitored across the entire 21-month trial period.

Logistic regression models will be used to establish and compare the ratios of positive musculoskeletal, functional, and falls-related outcomes in the four groups. An economic evaluation will also be carried out.

Participants in all groups will be invited to attend morning tea meetings at 3-monthly intervals. To build rapport and maximize retention, the meetings will have a primarily social function, but will also be opportunities to examine study diaries and encourage participants to continue to fill them out. Attendees will be instructed not to divulge or discuss group allocation at these meetings.

The VIBMOR trial will be reported according to CONSORT and the SPIRIT 2013 Checklist for randomized controlled trials (please see Additional File 1).

### Sample size

Bone loss occurs across the lifespan after the achievement of peak bone mass in young adulthood, an effect that is particularly marked in the years immediately post menopause. In our experience, roughly 30% of postmenopausal women of the age recruited for the current study do not lose hip aBMD over the course of a year [47]. In the absence of published data indicating the minimum percent improvement in the proportion of women losing bone over the course of a year required for public health benefit, we based our power calculations on a conservative estimate of 20%. Thus, from a two-sample difference of proportions test for 80% power with type 1 error of 5%, a sample size of 186 is required to detect hip aBMD maintenance or gain in 50% of the LIV group (30% + 20%). (To detect a greater improvement requires fewer participants, e.g., 30% difference requires, *n* = 84.) Our fully supervised, pilot study experienced a modest participant dropout of 6%. As a 2-year, home-based study could potentially experience greater attrition, we will over-recruit by 15% to power our secondary analyses. Thus, to test our primary hypothesis, we will recruit 214 participants (186 + 28). We therefore plan to recruit 428 participants, with 107 in each of the four arms (that is, LIV, HiRIT, LIV + HiRIT, and control).

We recognize a more traditional statistical approach is to compare actual change in aBMD between groups. We are reluctant to adopt the latter analysis as a primary approach because the absolute amount of change in hip aBMD that translates to a clinically significant effect has never been established. By contrast, it is well-recognized that prevention of any degree of age-related bone loss is highly clinically relevant if it prevents bone mass from falling below a fracture threshold. Thus, in our study population, maintenance or gain of total hip aBMD is the more justifiable clinical goal than absolute change in aBMD. Nevertheless, for the purposes of comparison with previous trials, we will perform both analyses and are powered to do so. To detect a 1.45% aBMD difference with 3.5% SD [48], at *α* = 0.05, for 80% power requires a group *n* of 92. 92 × 4 = 368 + 15% attrition allowance (13.8 × 4) = 423.2; essentially equivalent to our planned sample of 428. We will also perform per protocol analyses adjusting for compliance and any variable that differs between groups at baseline.

### Recruitment

Methods of recruitment will include advertising in print (magazines, and local community senior’s newsletters) and social media (Facebook and Twitter), radio and television interviews, and placing flyers in Medical Centers (endocrinologists and general practitioners) and senior citizens’ venues. Internal staff and student email broadcast calls for research volunteers within Griffith University will also be utilized. Volunteers may register interest on the dedicated trial website hosted by Menzies Health Institute Queensland, Griffith University, Australia (www.griffith.edu.au/vibmor). Recruitment for the VIBMOR trial started 1st June 2018 and will continue until June 2021.

### Eligibility and screening

#### Inclusion criteria

Participants must:

- be f emale
- be living in the community
- be generally healthy
- be postmenopausal (≥ 5 years postmenopause)
- weigh less than 125 kg (due to weight limit of LIV device)
- have aBMD of the FN, TH and/or LS in the osteopenic or osteoporotic range corresponding to a *T*-score ≤ − 1.0
- be willing and able to be allocated to any of the four trial arms.
- Either:
  - have been taking antiresorptive osteoporosis medications (e.g., hormone therapy, selective estrogen receptor modulator, bisphosphonate or denosumab) for a minimum of 12 months immediately prior to trial enrolment, or
  - not have been taking antiresorptive osteoporosis medications for a minimum of 12 months, or be osteoporosis treatment naïve,
  - AND not be intending to alter their osteoporosis medication status for the 21-month trial period.

#### Exclusion criteria

Volunteers will be excluded under any of the following conditions:

- Unable to stand or walk unaided
- Cognitive impairment
- Uncontrolled cardiovascular or respiratory disease
- Anabolic bone therapy (e.g., teriparatide, strontium ranelate, or romosozumab)
- Have undertaken moderate to high levels of bone-relevant physical activity in the immediately preceding twelve months (e.g., high-intensity resistance training)
- Regular use of vibration therapy in the preceding 12 months
- Malignancy
- Current or recent chemotherapy or radiation therapy
- Conditions known to adversely influence bone health (e.g., thyrotoxicosis, hyperparathyroidism, Paget’s disease, or renal disease)
- Currently taking other medication known to influence bone health (e.g., chronic corticosteroids, thiazides, or antiretroviral agents)
- Have sustained a fragility fracture in the past 6 months
- Have metal implants which prevent proximal femur or LS DXA scanning (e.g., bilateral total hip joint prostheses or lumbar spine fusion)
- Are unable or unwilling to take part in twice-weekly on-site supervised exercise training for 9 months.

There is no upper age limit for participants. Volunteers who contact the investigator will undergo a preliminary telephone screening. Prospective participants who are found to be eligible following the preliminary telephone screening will be provided with further information and a consent form and given adequate time to ask questions about the trial and consider participation. The prospective participant will then be invited to attend the Griffith University Bone Densitometry Research Laboratory for in-person screening and aBMD assessment (with DXA) to determine bone density at the proximal femur (TH and FN regions of interest) and LS (L1–L4). A participant will be eligible for inclusion if an aBMD *T*-score < − 1.0 is detected at any measured site.

All participants will be supplied with a full summary of their individual and overall study results after baseline testing and each subsequent assessment. Should any baseline aBMD results indicate evidence of severe osteoporosis, the individual will be referred to her primary care practitioner for review and be rescreened for eligibility thereafter.

Participation will be discontinued if a participant: (1) withdraws consent, (2) ceases to comply with allocated group activity for longer than 3 weeks, (3) initiates or discontinues osteoporosis medications, or initiates other medication known to adversely affect bone metabolism, (4) sustains an injury or illness external to trial activities which precludes further participation in their allocated activity, (5) initiates additional forms of bone-relevant exercise such as resistance training or impact-type exercise external to the trial, and/or (6) is advised by a general practitioner or medical professional to cease participation. There is a very low risk of injury during WBV and exercise; however, if a trial-related injury does occur the participant will be provided a discounted physiotherapy consultation at the Griffith University Allied Health Clinic. If further treatment is required, the participant will be referred to the appropriate healthcare professional. No other post-trial care or compensation for trial participation will be provided and is unlikely to be required. There is no other anticipated harm, and participants are free to withdraw at any time without penalty.

### Randomization and allocation

Once participant eligibility is established and the baseline assessment has been completed (i.e., the full suite of testing), eligible participants will be randomly assigned to a LIV, HiRIT, LIV + HiRIT, or control group. Randomization of eligible participants to one of the four groups will be performed using permuted blocks of random sizes, stratified by the presence (≥ 12 months exposure) or absence (lack of exposure) of osteoporosis medications for at least 12 months with a 1:1:1:1 allocation ratio. The allocation sequence has been prepared in advance under the supervision of the trial biostatistician (SKP) who is not located at Griffith University and therefore will not be involved in any participant-facing procedures such as recruitment, screening, testing, group allocation, or intervention delivery. The allocation sequence has been filed in sequentially numbered, opaque, sealed envelopes. A random subset (25%) of eligible participants will undergo blood collection for 25(OH)D and bone biomarker assessment at the three testing time points. Allocation concealment will be ensured by having the participant open the sealed envelope to reveal their allocation at the completion of their baseline assessment.

[Initially, group allocation was to be determined electronically using the web-based OpenClinica system, with the randomization scheme generated and managed by the VIBMOR trial lead biostatistician (SKP). Unfortunately, OpenClinica was taken offline in July 2020, forcing the randomization process to transition to a sealed envelope system.]

### Interventions

#### Low-intensity whole-body vibration (LIV)

The investigational LIV product to be examined in the current trial is the Marodyne LivMD platform, a LIV device manufactured by BTT Health GmbH (Inning am Ammersee, Germany) and distributed in Australia by RehaCare Pty Ltd (Cherrybrook, NSW, Australia). The Marodyne LivMD low-intensity vibration platform has been registered and approved as a medical device with the Therapeutic Goods Administration (Australian Register of Therapeutic Goods Certificate/ARTG identifier: 317506, Issued to RehaCare Pty Ltd). The Marodyne LivMD delivers a low-magnitude, vertical 30 Hz oscillation resulting in accelerations of 0.4*g* (where *g* is Earth’s gravitational field) which fall within safe exposure limits outlined by the International Standards Organisation for daily threshold limit values (ISO-2631) [49].

Trial staff will deliver and install LIV devices in the homes of participants allocated to LIV groups and perform training in its use. The LIV devices are programmed to operate for 10-min treatment epochs. Participants will be required to stand on the platform for one 10-min session, 5 days per week for nine months. Each device is equipped with an in-built electronic monitoring system that automatically records the date, time, and duration of device use. Transmissibility of the LIV stimulus to the lower appendicular and axial skeletal is inversely related to knee flexion angle [50] and positively related to straightness of stance [49]. Therefore, participants will be instructed to stand on the device with weight distributed evenly on each foot, knees extended, and trunk upright. Participants will receive a device operation manual and be instructed to contact the research team immediately should the device malfunction. A replacement device will be delivered to the participant promptly to avoid disruption of the intervention protocol. On completion of the 9-month period, the allocated device will be collected by trial staff or returned to the trial site by the participant. Following installation of the LIV platform, weekly calls will be made to participants for the first fortnight to check the device is operational and ensure sessions have been completed. Monthly emails will act as reminders to complete LIV diary entries and reinforce the required treatment exposure of five × 10 min per week of LIV therapy.

#### High-intensity resistance and impact training (HiRIT)

Participants allocated to HiRIT will attend two on-campus, supervised, 30-min exercise sessions per week on non-consecutive days for 9 months. The HiRIT protocol will be identical to the four exercises comprising the LIFTMOR trial protocol conducted in the same sample demographic, previously described [15]. An initial 2 to 4 weeks constitute a familiarization period in which participants will learn the exercise techniques through low-load variants. Thereafter, loading will be increased progressively to achieve lifting at ≥ 80–85% of 1 repetition max (RM) in five sets of five repetitions. The impact-loading exercise will also be performed in five sets of five repetitions. HiRIT sessions will be performed in small groups with a maximum of six participants per instructor. Training will be fully supervised by research staff who are at a minimum qualified Exercise Scientists, Exercise Physiologists or Physiotherapists. Attendance and weight progressions will be documented by the instructor in individual training diaries at every session, along with any adverse events.

### Control group activities

The control group will follow a low-intensity, home-based exercise program known to be an ineffective bone stimulus, but with possible balance and mobility benefits. An active control group was included to maximize participant retention and avoid ethical issues related to withholding all beneficial activities from individuals who may be at increased risk of osteoporotic fracture. The home-based, unsupervised, control regime will be comprised of two 30-min sessions per week on non-consecutive days, consisting of a 10-min walking warm-up, four stretches (neck, shoulder, calf, and lateral trunk flexion stretch) and low-resistance exercises with a focus on flexibility, lower limb muscular endurance, and balance, followed by a 5-min warm-down walk. The four low-resistance exercises can be selected from a battery of options to maintain variety (sit-to-stands, toe walks, alternating leg lunges, bilateral calf raises, standing forward shoulder raise, and shrugs). Intensity of the resistance exercises will be progressed mildly by increasing load (from no weight during the familiarization period, to 1 and 2 kg dumbbells, to a maximum of 3 kg) and increasing repetitions across the intervention period (to a maximum of three sets of 10 to 15 repetitions at ≤ 60% of one RM). A full set of dumbbells (2 × 1 kg, 2 × 2 kg, and 2 × 3 kg) and instructions on how to perform each stretch and exercise will be provided to participants.

Following baseline assessments, weekly calls will be made to participants for the first fortnight to confirm they have completed the twice-weekly sessions as prescribed. Monthly emails will act as reminders to complete home-based exercise program diary entries and reinforce the required treatment exposure of 2 × 30 min per week and maintain investigator contact.

### Outcome measures

All outcomes will be measured at baseline (T0), 9 (T1), and 21 (T2) months by investigators who are blind to group allocation at baseline (before randomization), using identical facilities, procedures, and equipment. All testing will take place in the Bone Densitometry Research Laboratory, Griffith University, Gold Coast campus, Queensland, Australia. A detailed summary of outcome measures is presented in Table 2.

**Table 2:** Summary of primary and secondary outcome measures and timing of collection; baseline (T0), 9 months (T1), 21 months (T2)

Variable	Data collection method	Data collection time points
**Primary outcome measure**		
Left total hip aBMD	DXA	T0, T1, T2
**Secondary outcome measures**		
Anthropometry	Height, weight, BMI, waist circumference	T0, T1, T2
Posture	Thoracic kyphosis angle, Tragus-to-wall	T0, T1, T2
Bone parameters	DXA left FN, LS and WB aBMD	T0, T1, T2
	3D DXA proximal femur trabecular and cortical bone mass and geometry	T0, T1, T2
	pQCT tibia and radius trabecular, total and cortical bone mass and biomechanical strength indices	T0, T1, T2
	QUS calcaneal BUA, SOS and SI	T0, T1, T2
Body composition	DXA WB lean mass, appendicular lean mass, fat mass and body fat %	T0, T1, T2
	pQCT leg and forearm muscle cross-sectional area and density	T0, T1, T2
Physical performance, strength, and balance	Timed up-and go; Five-times sit-to-stand; Leg extensor strength; Back extensor strength; Countermovement vertical jump; Tandem walk; Functional reach test	T0, T1, T2
Serum vitamin D and bone turnover markers	Blood collection	T0, T1, T2
Fall and fracture incidence, Safety (adverse events and injuries)	LIV/HiRIT/Control training diary entries	T0 → T1
	HiRIT trainer records	T0 → T1
	Monthly email correspondence	T0 → T2
	Detraining diary entries	T1 → T2
Physical activity enjoyment	Physical Activity Enjoyment Scale (PACES)	T0, T1, T2
Pelvic floor health and disability	Pelvic Floor Distress Inventory (PFDI-20); Pelvic Floor Impact Questionnaire (PFIQ-7)	T0, T1, T2
Health-related quality of life	Assessment of Quality of Life (AQoL-6D)	T0, T1, T2
Compliance monitoring	LIV/HiRIT/Control training diary entries	T0 → T1
	LIV internal compliance data download	T1
Economic evaluation	Healthcare utilization records	T0, T1, T2

Key:T0, baseline testing; T1, 9-month follow-up testing; T2, 21-month follow-up testing

#### Primary outcome

The primary outcome measure will be DXA-derived total hip aBMD (Norland ELITE, Norland at Swissray, Fort Atkinson, WI, USA).

#### Secondary outcomes

Secondary outcomes will include the following: anthropometric measures (height, weight, body mass index [BMI], and waist circumference); posture (inclinometer-determined thoracic kyphosis angle and tragus-to-wall); body composition (whole-body [WB] lean mass, fat mass, appendicular lean mass and percent body fat from DXA; leg and forearm muscle mass and density from peripheral Quantitative Computed Tomography [pQCT]); WB and regional bone mass and strength indices (FN, LS, and WB aBMD from DXA; FN geometry from 3D hip analysis of DXA scans; cortical and trabecular tibial and radial bone mass, strength and geometry from pQCT); physical performance and balance using a series of standardized tests (timed up-and-go, five-times sit-to-stand, functional reach test, leg extensor and back extensor maximal isometric muscle strength, lower extremity muscle power, and tandem walk test); quality of life; pelvic floor dysfunction and health; physical activity enjoyment; and bone turnover makers. Adverse events, fall and fracture incidence, and compliance will be tracked, and an economic evaluation conducted. Serum 25(OH)D, daily calcium consumption, and habitual bone-relevant physical activity will be determined in order to adjust for those variables in the analyses.

##### Anthropometric measures, body composition, and posture

Height will be measured to the nearest 0.1 cm, with the head positioned in the Frankfort plane, using a wall-mounted stadiometer (Model 216, Seca, Hamburg, Germany). Weight will be measured to the nearest 0.1 kg using a mechanical beam scale (Model 700, Seca, Hamburg, Germany). Height and weight will be measured without shoes and in light clothing. BMI will be calculated per the standard formula (BMI = weight/height^2^, kg/m^2^).

Waist circumference will be measured to the nearest 0.1 cm using a steel anthropometric tape at the mid-point between the lower margins of the most inferior ribs and the superior margin of the iliac crests on bare skin at the end of gentle expiration [51].

Lean mass (kg), fat mass (kg), percentage body fat (%), and appendicular lean mass (kg) will be derived from WB DXA (Norland at Swissray, Fort Atkinson, WI, USA; host software Version 4.7.4). Muscle cross-sectional area (cm^2^) and muscle density (mg/cm^3^) will be examined at the 66% site of the leg and forearm using pQCT (Stratec XCT-3000, Medizintechnick GmbH, Pforzheim, Germany). Coefficients of variation for pQCT-derived muscle cross-sectional area and density in our lab are 0.66% and 0.75% at the leg and 1.02% and 0.44% at the forearm, respectively.

Thoracic kyphosis will be assessed manually using a gravity-referenced inclinometer (Plurimeter, Australasian Medical & Therapeutic Instruments, Australia) following a procedure similar to that outlined by Harding and colleagues [18]. Thoracic kyphosis will be determined in two postures: [1] relaxed standing (neutral posture), and [2] standing “at attention.” After palpation and marking of relevant bony landmarks, the gravity-referenced inclinometer will be zeroed at the 7th cervical to 1st thoracic intervertebral space, and the angle (in degrees) at the 12th thoracic to 1st lumbar intervertebral space will be recorded. Duplicate measures of each posture will be performed, and the average will be used for analysis.

Tragus-to-wall distance, a measure of forward head posture, will be measured to the nearest 0.1 cm using an anthropometric ruler with the head in a natural position. The participant will be positioned in standing unshod with their feet shoulder-width apart, knees extended, with buttocks and back against the wall, and arms hanging relaxed by their sides. Horizontal distance (cm) from the anterior edge of the right tragus to the wall will be determined in duplicate, and the mean of the two attempts recorded.

##### Whole body and regional bone parameters

FN, anteroposterior LS (L1–L4), and WB aBMD (g/cm^2^) will be determined from DXA scans using the Norland ELITE (Norland at Swissray, Fort Atkinson, WI, USA; host software Version 4.7.4).

Bilateral proximal femur DXA scans (Medix DR, Medilink, Mauguio, France) will be conducted and analyzed using 3D Hip analysis software (DMS Group, Mauguio, France) to estimate trabecular and cortical bone mass and geometric parameters. The coefficient of variation for 3D Hip outcomes at the TH and FN in our laboratory range from 0.83 to 2.71%.

Calcaneal quantitative ultrasound (QUS; Achilles EXPII, GE Healthcare, Wisconsin, USA) of the functionally non-dominant limb will be performed to obtain bone strength parameters including broadband ultrasound attenuation (BUA; dB/MHz), speed of sound (SOS; m/s), and stiffness index (SI; %). We have previously observed that the skeletally non-dominant (lowest BUA) calcaneus corresponds to the functionally non-dominant leg (stance leg when kicking a ball) [52]. The coefficients of variation in our laboratory for BUA, SOS, and SI of the calcaneus in women over the age of 50 years range from 0.41 to 2.19%.

Bone strength parameters at the 4%, 38%, and 66% sites of the skeletally non-dominant leg and 4% and 66% sites of the skeletally non-dominant forearm (opposite to the hand they write with) will be scanned using pQCT (Stratec XCT-3000, Medizintechnick GmbH, Pforzheim, Germany). The distal 4% site for both the leg and forearm will be used to examine predominantly trabecular bone outcomes of the tibia and radius, respectively. The diaphyseal 38% site of the leg and the proximal 66% site of the forearm will be used to examine predominantly cortical bone outcomes of the tibia and radius, respectively. pQCT-derived bone parameters at the distal site will include total content (mg), total volumetric density (mg/cm^3^), total cross-sectional area (mm^2^), trabecular content (mg), trabecular volumetric density (mg/cm^3^), trabecular cross-sectional area (mm^2^), total bone strength index (g^2^/cm^4^), and trabecular bone strength index (g^2^/cm^4^). pQCT-derived bone parameters at the diaphyseal or proximal site will include: cortical content (mg), cortical volumetric density (mg/cm^3^), cortical cross-sectional area (mm^2^), cortical thickness (mm), periosteal circumference (mm), endocortical circumference (mm), polar section modulus (mm^2^), and polar strength strain index (mm^3^). Bone strength index reflects resistance of the bone to compression, and polar strength strain index reflects diaphyseal resistance to bending and torsional bone strength [53]. Scan procedures, scan parameters, and details outlining the technical loop analysis using host software (Version 6.20, Stratec, Medizintechnick GmbH, Pforzheim, Germany) have been described elsewhere [54]. In our laboratory, the coefficients of variation for pQCT-derived bone outcomes of the tibia range from 0.72 to 2.66% at the distal (4%) site and 0.21 to 1.38% at the diaphyseal (38%) site, and from 0.96 to 5.05% at the distal (4%) site and 0.61 to 3.27% proximal (66%) site for the radius.

All DXA, QUS, and pQCT scans will be performed by trained technicians following standardized scanning procedures and devices will be calibrated daily according to manufacturer quality control procedures. Analyses will be performed in accordance with manufacturer guidelines using host software.

##### Physical performance, strength, and balance

A series of commonly used, validated tests related to risk of falling will be used to assess physical performance, lower extremity isometric muscle strength, isometric back extensor muscle strength, lower extremity muscle power, and dynamic balance. Standardized instructions will be provided to each participant to maximize uniformity, followed by a demonstration of the task and a familiarization trial.

The timed up-and-go (TUG) test is a marker of functional mobility and dynamic balance [55]. Briefly, the participant will rise from a seated position without using their arms for assistance, walk as fast as possible without running a distance of 3 m, pivot at the indicated mark, and return to assume a seated position. The average time of two successful trials will be recorded.

The five-times sit-to-stand (FTSTS) test examines the ability to rise unassisted from a seated position and is a reliable marker of mobility and lower extremity muscle strength. The recommendations outlined by Bohannon for assessing older adults will be followed [56]. The average time of two successful trials will be recorded. Both the TUG and FTSTS tests will be performed using a standard chair (height = 45 cm) without arms.

Maximal lower extremity isometric muscle strength will be assessed using a leg strength dynamometer platform (TTM Muscle Meter, Tokyo, Japan) following the procedure outlined by Scott and colleagues [57]. Maximal isometric back extensor muscle strength will be determined using a dynamometer (Lafayette Instruments, Lafayette, IN, USA) following a previously validated method developed in our laboratory [58]. Both isometric strength measures will be performed in triplicate, and the maximal strength (kg) recorded.

Low leg muscle power is associated with low proximal femur aBMD and high fragility fracture risk in community-dwelling older women [59]. The countermovement vertical jump (CMVJ) will be performed to assess lower extremity muscle power by measuring maximal jump height, without arm swing, on a ground reaction force plate (Leonardo Mechanograph, Novotec Medical GmbH, Pforzheim, Germany). Participants will be provided with a description of the task, a demonstration, and one familiarization trial. Outcomes generated by host software (Version 4.4b01.32) for analysis will include maximal relative power during takeoff (W/kg), maximal velocity during takeoff (m/s), jump height (cm), maximal force (kN), relative maximal force (g), and Esslinger Fitness Index (%; comparison with age- and sex-matched reference norms of maximal power normalized to body weight). Technical details and testing procedures for the Leonardo Mechanograph have been published elsewhere [60], and the test-retest reliability in older adults is excellent (intra-class correlation coefficient = 0.93) [61].

Two clinical dynamic balance tests will be performed; the functional reach test (FRT) [62] and tandem walk [63] following published guidelines. For the FRT, the participant will stand upright with shoulders perpendicular to a wall marked with vertical measurement lines, with the right side closest to the wall, making a fist with the right hand. The right shoulder will be flexed until the right hand is level with shoulder height in front of the body, and the maximal distance (cm) reached by the head of the third metacarpal on the measurement lines will be noted. An instruction will then be given to reach forward as far as possible, by flexing the trunk at hip, but not taking a step forward or losing balance and the distance (cm) reached by the head of the third metacarpal will be measured again. Two FRT trials will be conducted and the maximum difference between the two measured distances will be recorded.

For the tandem walk test, the participant will be asked to walk heel-to-toe for 6 m along a straight line marked on the floor. They will be instructed to complete the tandem walk as quickly as possible, while making the least number of errors (such as, not touching heel to toe or stepping off the line to recover balance). Two trials will be performed; the trial with the least number of errors will be recorded. In the event that both trials have an equal number of errors, the time of the fastest trial (sec) will be recorded.

##### Serum 25(OH)D and bone turnover markers

A 5-mL blood sample will be collected by a trained phlebotomist from the antecubital vein in a random subsample of participants to determine serum 25(OH)D and bone turnover markers. After collection, blood will be centrifuged, and serum will be aliquoted and stored at − 80 °C until completion of the trial when samples will be analyzed using a single batch assay for the following: serum 25-hydroxyvitamin D, serum procollagen type-1 N-terminal propeptide (s-P1NP) and serum type 1 collagen cross-linked C-telopeptide (s-CTX). The bone turnover markers are classified as a bone formation marker (s-P1NP) and bone resorption marker (s-CTX).

##### Falls and fractures

For the purposes of the trial, a fall will be defined as “unintentionally coming to rest on the ground, floor or other lower level after an unexpected loss of balance which was not the result of a violent blow, loss of consciousness, sudden onset of paralysis or an epileptic seizure, and can include a slip or trip” [64]. Participants will be asked to record falls and fractures in their study diary and to call an investigator as soon as practically possible in order to fully describe the event and the consequences, including treatment and costs. Automated emails, requiring a reply to the investigators, in which participants are encouraged to promptly report the occurrence of a fall or fracture will be sent monthly to all participants during the entire trial period to serve as a reminder to do so. A falls and fracture case report form will be utilized to ensure consistently comprehensive details are recorded.

##### Pelvic floor dysfunction and health

Two validated condition-specific quality of life questionnaires related to pelvic floor dysfunction and health will be completed, the Pelvic Floor Distress Inventory short form (PFDI-20) and Pelvic Floor Impact Questionnaire short form (PFIQ-7), in order to track change in pelvic floor symptoms. These questionnaires were included as pelvic floor disorders (i.e., urinary incontinence and pelvic organ prolapse) are common in community-dwelling older Australian women [65] and there is anecdotal concern that heavy lifting may exacerbate the conditions. The PFDI-20 contains three subscales relating to symptoms of urinary, vaginal and bowel dysfunction, and the degree of bother and distress for women. The PFIQ-7 contains three subscales which assess the impact of pelvic floor disorders on activities, relationships, and feelings. The PFDI-20 and PFIQ-7 have been shown to have excellent test-retest reliability in older women (intra-class correlation coefficient = 0.93 and 0.77, respectively) [66]. Subscale and composite scores for each instrument will be calculated following official scoring rules.

##### Quality of life

The AQoL-6D [67], and instrument with high internal consistency (*α* = 0.76) [68], will be used to measure changes in wellbeing across the trial. The AQoL-6D is a self-administered brief 20 item questionnaire (taking 2–3 min) and is sensitive to small changes in the relatively high levels of wellbeing anticipated in the proposed cohort. The AQoL-6D will be scored using the AQoL-6D algorithm to obtain utility weights on a 0 = dead, 1 = full health scale. Population norms have been published using data from the 2007 National Survey of Mental Health and Wellbeing, a random sample of the entire Australian population [68]. The AQoL-6D also has six separately scored domains (independent living, mental health, coping, relationships, pain, and senses). Respondent AQoL-6D scores are generated using a standardized scoring algorithm.

##### Acceptability

Physical activity enjoyment has been identified as a potential determinant of exercise adherence. The eight-item, 7-point Likert scale, Physical Activity Enjoyment Scale (PACES) [69] will be used to examine level of physical activity enjoyment of all participants. Higher scores on the PACES instrument denote higher perceived enjoyment of physical activity.

##### Compliance and general monitoring

Prior to each supervised HiRIT session, participants will rate their level of muscle soreness on a 10-point visual analog scale, and note any alterations to their habitual diet, physical activity regime, health, or medications since their previous session in a purpose-designed HiRIT diary. They will also record sets, repetitions, and weight lifted for each exercise at each session, and records will be monitored by the instructor. Completion of 72 HiRIT sessions over the course of the 9-month intervention period will be defined as 100% compliance.

Although the LIV device electronically logs every vibration session, participants will be asked to also record the date of each LIV session in their study diary along with any alterations to their habitual diet, physical activity regime, health, or medications since their previous session. Completion of 180 sessions will be considered 100% compliance to LIV over the course of 9-month intervention period.

Every home-based exercise session will be logged in a purpose-designed control group diary, along with any alterations to their habitual diet, physical activity regime, health, or medications since their previous session. Completion of 72 sessions over the course of the 9-month intervention period will be defined as 100% compliance of control group activities.

During the second phase of the trial (12-month detraining period), all participants will be provided with the same detraining diary in which they are encouraged to record any alterations to their habitual diet, physical activity regime, health, or medications.

##### Adverse events

Adverse events occurring in any group, whether deemed to be directly associated with intervention activities or not, will be closely monitored and recorded by participants and study staff along with details of severity, treatment, and outcomes. Monthly emails will serve as reminders to participants to contact study staff should they suffer any injury or event during the trial period, including anything that may not appear to be directly related to study activities. The supervising coach will record any adverse effect that occurs during a HiRIT session. Any adverse effect from a study activity will be reported immediately to the ethics committee in line with ethical reporting requirements. As all interventions have been deemed low-risk and the higher-risk activity will be fully supervised, the approving Human Research Ethics Committee (HREC) did not require the establishment of a Data Safety Monitoring Committee. Instead, the HREC will act in a monitoring role.

##### Economic evaluation of healthcare resource utilization

The cost-effectiveness of lifestyle interventions for osteoporotic fracture prevention are not yet fully understood, having rarely been conducted [70]. A health system and societal cost-utility analysis of treatment with LIV, HiRIT, and LIV + HiRIT versus control will be undertaken using two methods. First, the quality of life data from the AQoL-6D will be used to transform into utility weights [67] and calculate quality-adjusted life-years (QALYs). Second, health service use data will be obtained from Queensland Health (for inpatient and outpatient procedures, costed using Diagnostic Related Groups - AR-DRGs v5.2). Participants will be asked to record health care utilization in their study diaries. Incremental cost-effectiveness ratios (ICERs) will be calculated by comparing intervention and control groups. Both a within trial analysis and a modeled (extrapolated over the rest of life) evaluation will be undertaken [71].

Participants will be issued a retrospective questionnaire at baseline to capture resource utilization in the previous 9 months prior to commencing their allocated group activities. Examples of details will be included to prompt participants to record the frequency, reason, and cost of items such as health care professional consultations (e.g., general practitioner, physiotherapist, podiatrist, endocrinologist), medication usage (including over the counter and prescription medications), medical aid purchases (e.g., orthotics), specialized laboratory tests and investigations (e.g., pathology and medical imaging), and inpatient and outpatient hospitalizations (emergency presentations and admissions for elective procedures). Purpose-designed prospective health care diaries will then be issued to participants in which they will be asked to record all healthcare resource utilization. The purpose-designed healthcare utilization diaries were implemented in the absence of any preexisting instrument to estimate total resource use, expenses, and lost productivity due to illness and/or injury [72]. To facilitate complete reporting of healthcare utilization, participants will be reminded in each monthly correspondence email and when attending seminars to complete their diaries to minimize inaccuracies and incomplete responses.

Data pertaining to emergency department presentations, hospital admissions, and procedures, including diagnostic scans will be obtained from Queensland Health records. Costs of prescription medications will be based on those available on the Australian Pharmaceutical Benefits Scheme and other costs that cannot be recalled will be determined from relevant schedules. Participants will additionally record any lost productivity and costs of care due to illness and treatment.

We will calculate the costs of delivering the twice-weekly home-based exercise program (i.e., the control group intervention), LIV, and HiRIT interventions for 9 months.

### Lifestyle behaviors and demographics

A number of lifestyle behaviors have the potential to influence bone outcomes, including diet and habitual physical activity and therefore may need to be adjusted for in the data analyses. Validated questionnaires will be used to determine average daily calcium consumption and historical bone-relevant physical activity.

Average daily calcium intake (mg/day) including supplementation will be assessed using the AusCal, a calcium-focused questionnaire designed for the Australia diet [73]. Questionnaire responses will be scored using FoodWorks analysis software (Version 7, Xyris Software, Brisbane, Australia).

Bone-relevant lifetime and current physical activity participation scores will be derived using the Bone-specific Physical Activity Questionnaire (BPAQ) [74], using a custom-designed online analysis program (www.fithdysign.com/BPAQ/). Participants will record all regular, structured physical activity, and the years of participation from which current (cBPAQ, i.e., previous 12-month period) and total (tBPAQ) scores will be calculated from algorithms that rank and weight activities based on loading rates and magnitudes.

Demographic and health data related to risk of fracture [44] will be collected at baseline, including ethnicity, smoking status (current, past, or never), alcohol consumption (average daily and weekly consumption), previous history of falls and low-trauma fractures, and parental history of hip fracture.

### Data integrity and dissemination

Management, storage, and retention of study data will be in line with Griffith University Code for the Responsible Conduct of Research. Data will be de-identified using unique coded IDs for each participant. Relevant study personnel will be trained in the use of the study database and assigned password access by the Primary Investigator for the purposes of data entry. All study-related documents and records will be retained securely for a minimum of 15 years after trial completion in a locked filing cabinet and on a password-protected hard drive within a secured office at Griffith University. De-identified data may be made available for the purposes of meta-analyses or other collaborations on a case-by-case basis; participant confidentiality will always be maintained. The usual scientific reporting practices will occur in order to disseminate trial results, for example scientific presentations and publication of papers in peer-reviewed discipline-specific journals. There will be no interim analyses published prior to completion of the VIBMOR trial.

### Blinding

The biostatistician responsible for data analyses will be blind to group allocation and have no contact with participants at any stage of the trial. The Primary Investigator will have some contact with participants (e.g., at morning teas and consulting on screening and data collection queries) but be blind to group allocation. The clinical trial coordinator and other research assistants will be blind to group allocation until after baseline testing when randomization will occur and thereafter will be aware of group allocations. Participants will be blind to group allocation until after baseline testing. Although they will naturally be aware of allocation to their respective group (that is, LIV, HiRIT, LIV plus HiRIT, or home-based exercise [referred to herein as control]), they will remain blinded to the study hypotheses. That is, the group activity hypothesized to be most efficacious will not be revealed to participants.

### Data analyses

Intention-to-treat analyses will be performed, i.e., all participants regardless of compliance or attrition, will be included in the final analyses to compare the proportion of participants maintaining TH aBMD between groups. At 9 months, separate analyses of the two primary hypotheses (LIV with and without HiRIT) will be performed. To compare proportions of participants maintaining TH aBMD across treatment groups in both primary and secondary hypotheses, logistic regression models will be used, with adjustments for age, baseline values, BMI, bone-relevant physical activity, calcium consumption, and serum 25(OH)D. Repeated measures comparisons of mean change in TH aBMD and secondary outcomes will also be conducted using univariate analyses, controlling for initial values, calcium, serum 25(OH)D, and compliance. Separate per protocol analyses based on adherence to exercise and LIV will be conducted using treatment log diaries and information obtained during study visits. The change in quality of life scores over time will be compared between study groups using appropriate parametric and non-parametric techniques. Further exploratory analyses will include examination of secondary hypotheses and possible associations of osteoporosis medications, as well as anthropometric and compliance factors, on treatment effects. Repeat analyses of the 21-month data will be conducted to examine whether any treatment effects have been maintained.

### Roles and responsibilities

The coordinating center will be the Bone Densitometry Research Laboratory in the Menzies Health Institute, Griffith University, Gold Coast, Queensland, where all face-to-face trial activities will be conducted. Responsibility for all aspects of trial conduct and local organization will be overseen and monitored by the primary investigator (BRB) with contributions from co-investigators (CTR, SKP, and MF) as required. Implementation of the trial protocol will be the responsibility of the clinical trial coordinator (ATH), with appropriate delegation to research assistants. No additional trial steering committee, endpoint adjudication committee, or Data Monitoring Committee was deemed necessary for reasons described below. Regular meetings of the clinical trial coordinator, research assistants, and primary investigator (BRB) will be held to review protocol adherence and safety reports. Research staff will be responsible for identifying potential recruitment channels (as outlined in the “Recruitment” section of the “Methods”), contacting potential participants to conduct the preliminary screening, and obtaining informed consent prior to baseline DXA screening and testing.

Only one mild adverse event was reported during the LIFTMOR trial (Protocol number AHS/07/14/HREC, ANZCTR trial number 12616000475448) that examined the identical HiRIT protocol in participants with identical eligibility criteria [15], providing evidence of a very low-risk intervention. The VIBMOR trial was therefore also deemed “low risk” by the approving HREC, and early termination was considered exceedingly unlikely. For this reason, stopping guidelines and a Data Safety Monitoring Board (DSMB) were determined to be unnecessary. Instead, in accordance with the Australian Code for the Responsible Conduct of Research developed by the National Health and Medical Research Council, and the University Code for the Responsible Conduct of Research, any adverse events that occur during the trial period that are possibly, likely, or certainly related to procedures/interventions will be reported to the HREC within 24 h for the consideration of the Chair. Further, any reported adverse events will be reviewed and adjudicated at the Griffith University HREC meeting held monthly. Compulsory annual progress reports will also be made to the HREC which include reporting of adverse events. The Griffith University HREC is a registered institution with the National Health and Medical Research Council (Registration number EC00162). No supplementary auditing for the trial was deemed necessary.

We do not anticipate that there will be any further protocol amendments to the VIBMOR trial; however, in the unlikely event that amendments are required, any variations will be submitted for approval by the Griffith University HREC and the funding body as required. Modifications will be updated on the ANZCTR clinical trials registry by the primary investigator (BRB).

## Discussion

To our knowledge, VIBMOR will be the first trial to examine the efficacy of LIV alone or in combination with a bone-targeted HiRIT program on determinants of hip fracture in postmenopausal women with low to very low bone mass. The overarching aim of the VIBMOR trial is to compare the effects of two non-pharmacological therapeutic strategies alone and combined, having previously only been applied in isolation. Pharmacotherapy is the currently accepted primary therapy for the prevention of osteoporotic fracture [5]. However, osteoporosis medications are not universally effective or broadly adopted, nor do they reduce falls in older women, despite falls being strongly associated with fragility fracture [75]. A therapy that both builds bone and reduces falls would be a valuable step forward in the prevention of osteoporotic fracture, with associated morbidity and mortality benefits.

Low-intensity whole-body vibration (LIV; 30 Hz at 0.3 to 0.4*g*) has been shown to stimulate meaningful bone benefits in children with clinical conditions [29–31] and adults with low BMD [34], osteoporosis [32], and chronic obstructive pulmonary disease [33]. Nevertheless, systematic reviews and meta-analyses report inconsistent effects of vibration on bone mass and strength specifically in postmenopausal women. Some report osteogenic effects, mainly at the LS [36, 76, 77]. A more recent review reports no overall effect on aBMD or bone microarchitecture, but a potential effect on falls [37]. The variability of outcomes reported in previous WBV reviews may be due to methodological shortcomings of some included trials. Those include inadequate sample size [78, 79], confounding simultaneous interventions (e.g., osteoporosis medications or vitamin D and/or calcium supplementation) [78, 80–84], lack of control group [78], inadequate blinding [78, 85], and/or insufficient trial duration [81]. Authors of reviews agree that large-scale randomized controlled trials of adequate duration are needed before recommendations can be made for the use of vibration as adjuvant osteoporosis therapy [36, 76, 77]. Trials examining bone mass and strength outcomes have compared WBV to very low-intensity aerobic activities unlikely to be osteogenic (walking) [79], to sham WBV [32, 86], or compared non-equivalent WBV stimuli (e.g., high- versus low-intensity WBV therapy [47]), in community-dwelling postmenopausal women. Few studies have examined the synergist effect of vibration and exercise [39], or directly compared WBV with exercise [38] in postmenopausal women, using appropriately robust randomized controlled trial design.

In a 6-month randomized controlled pilot trial of thrice-weekly WBV versus thrice-weekly resistance training (machine-based leg extension and leg press exercises) or control (habitual activity) for postmenopausal women, only WBV increased TH aBMD (0.93%, *p* = 0.03) [38]. The WBV group, however, was contaminated by the addition of static and dynamic knee extensor exercises during the vibration stimulus and the vibration protocol changed across the trial period (session duration, amplitude [from 1.7 mm to 2.5 mm], and/or frequency [from 35 to 40 Hz] were all increased) limiting the ability to identify the effective therapeutic protocol. Furthermore, the resistance training intensity in the exercise group ranged from only low intensity (20 repetition maximum) to moderate intensity (eight repetition maximum) which may have limited its osteogenic potential [38].

In the “Erlangen Longitudinal Vibration Study,” no difference in effect in LS aBMD was observed after 18 months of 25–35 Hz, amplitude 1.7 mm WBV plus exercise versus exercise alone (1.5 ± 2.3% versus 2.1 ± 3.0%, respectively) [39]. As previously discussed, the low-moderate-intensity nature of the exercise group was likely an insufficient stimulus for notable bone adaptation. Although there was no change detected in TH aBMD for either group, and findings did not support an additive effect for WBV plus exercise on bone mass, a promising reduction in fall incidence across the trial period for WBV plus exercise was observed in comparison to control (a wellness-focused program). As a vibration displacement of 1.7 mm at 30 Hz produces a stimulus intensity of 6.1 *g*, neither of the latter vibration protocols may be safe for an osteoporotic demographic.

Current exercise prescription recommendations support the notion that exercise should be performed to ameliorate age-related loss of bone mass in healthy older adults; however, there is disparity in the specifics of the recommendations. The “Too Fit to Fracture” exercise recommendations for individuals with reduced bone mass or osteoporotic vertebral fractures advocate only moderate-intensity progressive resistance training in combination with high challenge balance exercises and aerobic exercise for general health benefits [87]. The “Exercise & Sports Science Australia” position statement on exercise prescription for osteoporosis suggests impact-loading exercises, moderate-high-intensity progressive resistance training, and challenging balance activities to reduce fall and fracture susceptibility [88]. Findings of past meta-analyses have suggested resistance training, or a combination of resistance training and impact-type activities result in positive, albeit modest, effects on aBMD at clinically relevant sites [89, 90]. A recent very comprehensive systematic review revealed that most studies investigating the effects of exercise intervention on bone mass have applied insufficient loading to be osteogenic [91]. A recent meta-analysis of 53 randomized controlled trials in healthy postmenopausal women that parsed out the effect of exercise intensity on aBMD reported that high-intensity exercise improved LS aBMD more than low or moderate-intensity exercise [13]. A dearth of trials (only 3) testing high-intensity exercise may have limited the ability to detect a similar effect at the proximal femur.

Previously, it was thought that individuals with low bone mass could not tolerate high magnitudes and rates of loading on account of their weakened skeleton. This assumption was discredited by findings of the LIFTMOR and LIFTMOR-M intervention trials that reported improvements in LS and FN aBMD, functional performance, muscle strength, balance [15], and thoracic kyphosis [17], with no fragility fractures at any skeletal site during the intervention period [15–18]. These trials showed that, given adequate supervision, and a familiarization period, HiRIT is safe, well tolerated, and feasible for individuals at increased risk of fragility fracture. Moreover, there has been concern among the research community that high-intensity resistance training may worsen symptoms of pelvic floor disorders or lead to pelvic floor dysfunction in older women; however, recent findings from an Australian cross-sectional study do not support this notion [92]. It was reported that women aged 18 to 88 years who lifted heavy weights for exercise did not have an increased prevalence of symptoms of pelvic organ prolapse, but rather those who were inactive or lifted *lighter* weights for exercise presented with an increase in pelvic floor organ prolapse symptoms.

The promising results from LIV and HiRIT trials suggest there is reason for optimism that non-pharmacological therapies may be legitimate options for reducing falls and fragility fractures in older adults with low bone mass. To date the synergistic effect of LIV with HiRIT has never been tested, nor has there been a direct comparison of efficacy between LIV and HiRIT for reducing risk of osteoporotic fracture. The VIBMOR trial will not only examine these important research questions, but include both women who are on or off osteoporosis medications, and women with low to very low bone mass, populations who have traditionally been excluded from intervention trials testing non-pharmacological osteoporosis therapy [91].

### Limitations

Several limitations warrant discussion. First, the VIBMOR trial will not be powered to detect differences in falls and fracture incidence between the four trial arms. Second, for reasons of participant retention, we elected to adopt a positive control condition (home-based low-intensity exercise program) such that the control arm of the trial will not be entirely exercise naïve. When volunteering under the expectation of receiving therapy for osteoporosis, arguably there are ethical issues around withholding all forms of potentially beneficial therapy from individuals at risk of fragility fracture. While the home-based exercise program is not expected to provide a stimulus for bone it may provide some protection from falls. Nevertheless, it is acknowledged the control group intervention also differs from the HiRIT intervention in terms of weekly face-to-face contact with investigators. We will address this with frequent electronic contact and morning tea meetings. Third, our sample will be limited to apparently healthy, ambulatory, community-dwelling postmenopausal women with low to very low bone mass. These inclusion criteria will limit generalizability of results to men and women who may be even more frail and therefore even more in need of therapy designed to prevent osteoporotic fracture.

## Conclusion

The growing worldwide burden of osteoporotic fracture and lack of appetite of patients for osteoporosis medications demands that effective non-pharmacological therapies to prevent osteoporotic fractures are identified. The proposed study seeks to establish the efficacy of LIV alone and in combination with HiRIT, as two such therapies. A comprehensive suite of secondary and exploratory outcome measures will inform other benefits of either intervention such as body composition, posture, functional performance, and numerous indices of quality of life. An economic evaluation will add vital information as to the feasibility of the therapies as legitimate public health interventions.

## Trial status

Protocol version number 1.0, 26/05/2021, Protocol version number 2.0, 02/12/2021

Recruitment initiated 01/06/2018 and approx. recruitment completion date 30/06/2021.

## Supplementary Information

**Additional file 1.** Populated SPIRIT 2013 Checklist.

## Data Availability

The final trial dataset will be accessible.

## Abbreviations

aBMD: Areal bone mineral density
BMD: Bone mineral density
BPAQ: Bone-specific Physical Activity Questionnaire
BUA: Broadband Ultrasound Attenuation
cBPAQ: Current Bone-specific Physical Activity Questionnaire score
CONSORT: Consolidated Standards of Reporting Trials
DSMB: Data Safety Monitoring Board
DXA: Dual-energy X-ray Absorptiometry
FN: Femoral neck
FRT: Functional reach test
FTSTS: Five-times sit-to-stand
HiRIT: High-intensity resistance and impact training
HREC: Human Research Ethics Committee
ICERs: Incremental cost-effectiveness ratios
LIV: Low-intensity whole-body vibration
LS: Lumbar spine
PACES: Physical Activity Enjoyment Scale
PFDI-20: Pelvic Floor Distress Inventory
PFIQ-7: Pelvic Floor Impact Questionnaire
pQCT: Peripheral Quantitative Computed Tomography
QALYs: Quality-adjusted life-years
QUS: Quantitative Ultrasonometry
SI: Stiffness Index
SOS: Speed of sound
SPIRIT: Standard Protocol Items: Recommendations for Intervention Trials
s-CTX: Type 1 collagen cross-linked C-telopeptide
s-P1NP: Procollagen type-1 N-terminal propeptide
tBPAQ: Total Bone-specific Physical Activity Questionnaire score
TH: Total hip
TUG: Timed up-and-go
T0: Baseline
T1: 9-month follow-up
T2: 21-month follow-up
VIBMOR: Vibration and exercise Intervention for Bone, Muscle and Osteoporosis Rehabilitation
WB: Whole body
WBV: Whole-body vibration
25(OH)D: Serum 25-hydroxyvitamin D
